# Utilizing CRISPR-Cas13d-knockdown in zebrafish to study a rare monogenic bone fragility syndrome

**DOI:** 10.1093/jbmrpl/ziaf153

**Published:** 2025-10-23

**Authors:** Kirsi Määttä, Yu-Chia Chen, Sandra Pihlström, Riikka E Mäkitie, Emilie Dambroise, Laurence Legeai-Mallet, Pertti Panula, Outi Mäkitie, Minna Pekkinen

**Affiliations:** Folkhälsan Research Center, Genetics Research Program, 00290 Helsinki, Finland; Research Program for Clinical and Molecular Metabolism, Faculty of Medicine, 00014 University of Helsinki, Helsinki, Finland; Department of Anatomy, 00014 University of Helsinki, Helsinki, Finland; Zebrafish Unit, HILIFE, 00014 University of Helsinki, Helsinki, Finland; Folkhälsan Research Center, Genetics Research Program, 00290 Helsinki, Finland; Research Program for Clinical and Molecular Metabolism, Faculty of Medicine, 00014 University of Helsinki, Helsinki, Finland; Folkhälsan Research Center, Genetics Research Program, 00290 Helsinki, Finland; Research Program for Clinical and Molecular Metabolism, Faculty of Medicine, 00014 University of Helsinki, Helsinki, Finland; Department of Otorhinolaryngology–Head and Neck Surgery, Helsinki University Hospital and University of Helsinki, 00014 University of Helsinki, Helsinki, Finland; Laboratory of Genetics of Developmental Disorders, INSERM UMR 1163, Université Paris Cité, Imagine Institute, 75015 Paris, France; Laboratory of Genetics of Developmental Disorders, INSERM UMR 1163, Université Paris Cité, Imagine Institute, 75015 Paris, France; Department of Anatomy, 00014 University of Helsinki, Helsinki, Finland; Folkhälsan Research Center, Genetics Research Program, 00290 Helsinki, Finland; Research Program for Clinical and Molecular Metabolism, Faculty of Medicine, 00014 University of Helsinki, Helsinki, Finland; Children's Hospital, University of Helsinki and Helsinki University Hospital, 00029 HUS, Helsinki, Finland; Department of Molecular Medicine and Surgery and Center for Molecular Medicine, Karolinska Institutet, 171 77 Stockholm, Sweden; Department of Clinical Genetics, Karolinska University Hospital, 171 77 Stockholm, Sweden; Folkhälsan Research Center, Genetics Research Program, 00290 Helsinki, Finland; Research Program for Clinical and Molecular Metabolism, Faculty of Medicine, 00014 University of Helsinki, Helsinki, Finland; Children's Hospital, University of Helsinki and Helsinki University Hospital, 00029 HUS, Helsinki, Finland

**Keywords:** sphingomyelin synthase 2, sphingomyelin metabolism, early-onset osteoporosis, calvarial doughnut lesions with bone fragility, zebrafish

## Abstract

Pathogenic variants in the human *SGMS2* gene coding for SMS2 protein result in a rare form of primary osteoporosis, “Calvarial doughnut lesions with bone fragility” (CDL). To obtain a better understanding of the *SGMS2* gene’s function and the molecular mechanisms underlying CDL, we studied mRNA expression of the zebrafish orthologs for *SGMS2*, *sgms2a* and *sgms2b*, in WT zebrafish and developed zebrafish models using the novel CRISPR-Cas13d-knockdown. The *sgms2a*, *sgms2b*, and *sgms2a+b* knockdown zebrafish were analyzed by embryo phenotype at 2 d post-fertilization and cartilage and bone staining at 7 d post-fertilization. In situ hybridization studies of embryonic and early larval WT zebrafish demonstrated *sgms2a* expression in the brain, myotome, and craniofacial skeletal and cartilage elements, and *sgms2b* expression in the myotome and craniofacial cartilage elements. Single-cell RNA sequencing of juvenile WT zebrafish calvaria cells detected high *sgms2a* expression in osteogenic cells. Knockdown of *sgms2a* and *sgms2b* in zebrafish had detrimental effect on embryonic development and compromised notochord and craniofacial formation. Our findings provide novel information on the role of *SGMS2* in the musculoskeletal system. The CRISPR-Cas13d-knockdown zebrafish models for CDL serve as preliminary platforms for exploring embryonic and early larval gene function and obtaining clues for molecular mechanisms underlying its pathology.

## Graphical Abstract

**Graphical Abstract:**
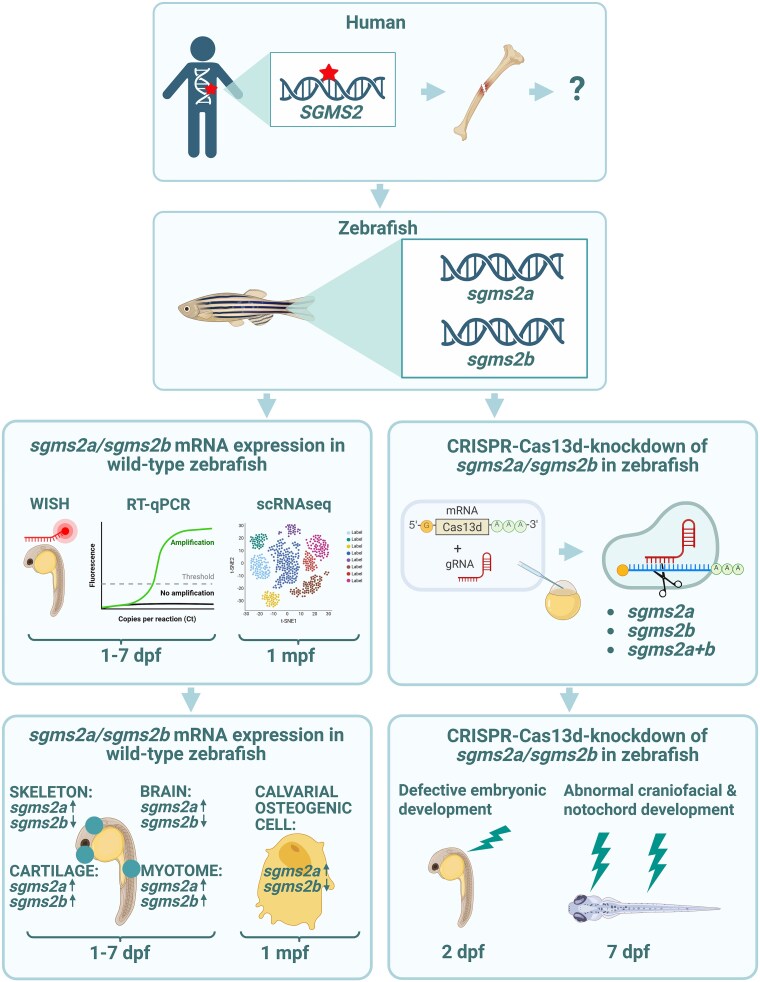

## Introduction

Osteoporosis (OP) is characterized by decreased and microarchitecturally deteriorated bone tissue, and consequently increased skeletal fragility.^1^ Osteoporosis is a multifactorial disease with a strong genetic component.^2^ While OP is considered a polygenic condition, on rare occasions single gene variants may result in early-onset OP.^3^ One such newly discovered monogenic form is the Calvarial doughnut lesions with bone fragility disorder (CDL; OMIM #126550) arising from heterozygous variants in sphingomyelin synthase 2 gene (*SGMS2*).^4^ Calvarial doughnut lesions with bone fragility is characterized by low BMD, multiple spinal and peripheral fractures since childhood, and sclerotic doughnut-shaped lesions in cranial bones.^4^ More severely affected individuals exhibit neonatal fractures, severe short stature, marked cranial sclerosis, and spondylometaphyseal dysplasia.^4^ In addition, neurological manifestations, including recurrent facial or other cranial nerve palsies, may occur.^4^

Sphingomyelin synthases (SMSs), encoded by sphingomyelin synthase genes, play a central role in the sphingolipid biosynthetic pathway by producing sphingomyelin, the most abundant sphingolipid and a significant component of the cell membrane.^5^ Its two catalytically active isoforms, SMS1 and SMS2, both catalyze the same reaction but have different subcellular localizations; SMS1 is localized in the Golgi, and SMS2 resides mainly in the plasma membrane.^5^ While recent studies have shown SMSs to have a critical role in skeletal and neural tissues,^6^ the key molecular functions remain incompletely understood.

Prior studies have used animal models to explore sphingolipid pathway-related mechanisms underlying musculoskeletal pathologies. Matsumoto et al. reported that a mouse with a deficiency in SMS1 but not SMS2 had reduced bone formation.^7^ SMS1 regulated osteoblast development in neonatal and postnatal bone formation through BMP2 signaling.^7^ Stoffel et al. and Aubin et al. reported that mouse models lacking functional sphingomyelin phosphodiesterase 3 (SMPD3), an enzyme that further processes sphingomyelin, displayed skeletal deformities.^8^^,^^9^ Interestingly, while *Sgms2* KO mice have also been used in metabolic studies, no apparent skeletal abnormalities have been observed.^10^^,^^11^ To overcome this major limitation, a lack of skeletal phenotype, of the *Sgms2* deficient mouse as a model for CDL, zebrafish (*Danio rerio*) has emerged as an attractive alternative.

Zebrafish (*D. rerio*) have become an important vertebrate model organism to study human skeletal diseases owing to strong similarities in their skeletal physiology to mammals, external development, transparency of embryos, and easy genetic manipulation.^12^^,^^13^ Compared to the human reference genome, approximately 71% of all human genes and most importantly, 82% of morbid human genes, have at least one zebrafish ortholog.^14^ In the case of *SGMS2*, zebrafish has two orthologs: *sgms2a* (ZFIN ID: ZDB-GENE-070730-2) and *sgms2b* (ZFIN ID: ZDB-GENE-040801-162)*.* Both gene orthologues are located on different chromosomes (*sgms2a:* chromosome 1; *sgms2b*: chromosome 23) and display several different transcript variants (*sgms2a*: four transcript variants; *sgms2b*: two transcript variants). In comparison, human *SGMS2* (ENSEMBL ID: ENSG00000164023) is located on chromosome 4 and displays 22 different transcript variants.

CRISPR-Cas9 is a widely used but relatively time-consuming genome-editing tool to create stable mutant lines in zebrafish.^12^^,^^13^ Recently, CRISPR-Cas13d emerged as an efficient knockdown (kd) platform to explore gene function in animal embryos by targeting both zygotically expressed and maternally provided transcripts.^15^ The method provides swift access to explore the outcomes of disrupted gene functions before access to the stable zebrafish mutant lines. The novel CRISPR-Cas13d platform is also a strong rival for commonly used knockdown methods, such as morpholinos, that often face challenges related to the induction of nonspecific effects, poor correlation between morpholino-induced and mutant phenotypes, as well as requirements of multiple proper controls and rescue experiments.^15–18^

This study investigated the functional role of the human *SGMS2* gene orthologs in zebrafish. First, we studied the expression pattern of both *sgms2a* and *sgms2b* mRNA during the early development days in WT whole-body zebrafish embryos and larvae by whole-mount in situ hybridization (WISH) and RT-qPCR, and in juvenile WT zebrafish calvaria cells by single-cell RNA sequencing. Second, we utilized the novel CRISPR-Cas13d-method to knock down the mRNA expression of *sgms2a* and *sgms2b* separately and together. The kd models were analyzed for phenotypic features at 2 d post-fertilization (dpf) and for cartilage and bone elements at 7 dpf. This is the first study to provide information on zebrafish *sgms2a* and *sgms2b* gene expression and function and to establish a novel zebrafish model for CDL with notochord and craniofacial abnormalities.

## Materials and methods

### Zebrafish strain and maintenance

Zebrafish of the WT AB strain were obtained from the Zebrafish unit, University of Helsinki.^19^^,^^20^ The zebrafish line was maintained under standard conditions, as described previously.^19^^,^^20^ Embryos were staged, according to Kimmel et al.^21^ CRISPR-Cas13d-transcriptome edited zebrafish targeting *sgms2a*, *sgms2b*, and *sgms2a+b* were generated in the WT AB zebrafish strain. All experiments were performed under standard conditions under institutional (University of Helsinki) and national ethical and animal welfare guidelines and regulations. All experimental procedures were approved by the Finnish Project Authorization Board (protocol No. ESAVI/43079/2019 and ESAVI/124/2023).

### *sgms2a* and *sgms2b* mRNA expression in WT zebrafish embryos and larvae by whole-mount in situ hybridization

Whole-mount in situ hybridization protocol by Thisse and Thisse^22^ was followed with modifications. The protocol was adapted for older embryos (3 and 6 dpf) by increasing the incubation time with anti-DIG antibody alkaline phosphatase (24 h) and proteinase K treatment time (35 min). The instructions provided by manufacturers were followed in each experiment. To prepare WISH probes, 40 WT zebrafish embryos at 1 dpf were sacrificed on ice, and total RNA extraction was performed using the RNeasy Mini kit (Qiagen). A tissue homogenization step was performed using 2 mL Lysing Matrix E tubes (MP Biomedicals) and SpeedMill PLUS homogenization system (Analytik Jena GmbH+Co KG) with 3 × 20 s homogenization. Up to 1 μg of total RNA was utilized for cDNA synthesis using QuantiTect Reverse Transcription kit (Qiagen). Primers for *sgms2a* and *sgms2b*, designed with Primer-BLAST,^23^ are reported in Table 1. PCR amplification was performed using Dream Taq DNA polymerase or DyNAzyme II DNA polymerase kit (Thermo Fisher). The PCR products were cloned into vectors using pGEM-T Easy Vector System and JM109 Competent Cells (Promega Corporation). Minipreps were prepared, and plasmid DNA purification was performed using QIAprep Miniprep Kit (Qiagen). Insert sequences were confirmed using BigDye Terminator v3.1 Cycle Sequencing Kit (Applied Biosystems) at the Sequencing unit of the Institute for Molecular Medicine Finland FIMM Technology Centre, University of Helsinki (supported by Biocenter Finland). *NcoI*, *SalI*, and *NdeI* (Thermo Fisher) enzymes were utilized for plasmid linearization. Linearized plasmids were purified using Wizard SV Gel and PCR Clean-Up System (Promega Corporation). In vitro transcription reactions (sense and antisense RNA probes) were carried out using T7 and SP6 RNA polymerases (Promega Corporation), DIG RNA labeling mix (Roche), and 1 μg of the linearized plasmid. Synthesized RNA probes were purified using MEGAclear Transcription Clean-Up Kit (Thermo Fisher) or Lithium Chloride Precipitation Solution (Invitrogen). For WISH, embryos were collected at 1, 2, 3, and 6 dpf (5-10 embryos per time point). Embryos were placed in 1.5 mL tubes during the wash and incubation steps, and solutions were added and removed from the tube using a Pasteur pipette. The WISH experiment was performed twice. Negative control (sense probe) data is presented in the Figure S1. Zebrafish were imaged with a DM IRB microscope equipped with Leica DFC490 camera (Leica Microsystems) using viewing chambers.^24^

**Table 1:** Primer and CRISPR-Cas13d gRNA sequences.

**Name**	**Sequence 5′>3′**	**Product length (bp)**
WISH		
*sgms2a* forward	GAATGTGGAGGGATGAAGGC	897
*sgms2a* reverse	GAGGTACGTGAGGGTGAGGATG	
*sgms2b* forward	CCACGACAGAGTCCCGGATA	756
*sgms2b* reverse	TAGCTCCTGCACGGGTTCTT	
RT-qPCR		
*sgms2a* forward	GTTGCTCTTTCACAGATACAAGGCTATT	207
*sgms2a* reverse	ATTGATAATCCACCGCCAGACA	
*sgms2b* forward	GAAGCTGCAGCGTGCTTATC	315
*sgms2b* reverse	CTCAAAGCCTGGTTGTTGGC	
CRISPR-Cas13d-knockdown		
Cas13d_Universal_t7	TAATACGACTCACTATAGGAACCCCTACCAACTGGTCGGGGTTTGAAAC	-
Cas13d_*sgms2a*_1	ACGTGCCTTGTTCTTTCTGCTGGGTTTCAAACCCCGACCAGTT	-
Cas13d_*sgms2a*_2	TCTGGCGGTGGATTATCAATCAAGTTTCAAACCCCGACCAGTT	-
Cas13d_*sgms2a*_3	GGTGGTACCATCTTTTGTGTTGGGTTTCAAACCCCGACCAGTT	-
Cas13d_*sgms2b*_1	GTGTTCGGCATGATATTAGTGGTGTTTCAAACCCCGACCAGTT	-
Cas13d_*sgms2b*_2	CGGCTCTGGACTGTTTATCATGTGTTTCAAACCCCGACCAGTT	-
Cas13d_*sgms2b*_3	TGTGGTGGAACTTCATGTTTTCCGTTTCAAACCCCGACCAGTT	-

Reference sequences: *sgms2a*, NM_001145563; *sgms2b*, NM_001003641.

### *sgms2a* and *sgms2b* mRNA expression in WT whole-body zebrafish embryos and larvae by RT-qPCR

Messenger RNA expression levels of *sgms2a* and *sgms2b* were analyzed in WT zebrafish. To isolate total RNA, larval whole-body zebrafish were sacrificed on ice at 1, 2, 3, 5, and 7 dpf, and 10-40 larvae were pooled together per sample per time point. Three replicate samples were prepared at each time point. Total RNA extraction was performed using the RNeasy Mini kit (Qiagen). A larval tissue homogenization step was performed using 2 mL Lysing Matrix D tubes (MP Biomedicals) and SpeedMill PLUS homogenization system (Analytik Jena GmbH+Co KG). RNA quantity was determined by a NanoDrop spectrophotometer (Thermo Fisher) and RNA quality by agarose gel electrophoresis. Up to 1 μg of total RNA was utilized for cDNA synthesis using QuantiTect Reverse Transcription kit (Qiagen). Primers for *sgms2a* and *sgms2b* were designed with Primer-BLAST^23^ and sequences are presented in Table 1. *rpl13* gene was used as a reference gene; primer sequences have been reported previously.^25^ RT-qPCR was carried out with the PowerUp SYBR Green Master Mix (Life Technologies) and the Bio-Rad CFX96 Touch Real-Time PCR Detection System (Bio-Rad Laboratories). Cycling parameters were the following: 50 °C for 2 min, 95 °C for 2 min, 40 cycles of 95 °C for 15 s, and 60 °C for 60 s. Melting curve analysis was performed at the end of cycles to ensure that only a single amplicon was obtained. All reactions were performed in four replicates. Results were evaluated using Bio-Rad CFX Maestro Software (Bio-Rad Laboratories). Relative gene expression was quantified using the delta delta Ct method.^26^

### *sgms2a* and *sgms2b* mRNA expression in juvenile zebrafish calvaria cells by single-cell RNA sequencing

The data of *sgms2a* and *sgms2b* in osteogenic cells from juvenile 1-mo-old zebrafish cranial vault were extracted from the single-cell RNA sequencing analyses performed by Dambroise et al., using the Seurat R package (version 4.1.3) and the standard Seurat v3 integration workflow^27^^,^^28^ using the same filters ((1) unique feature counts over 2000 or below 500 or (2) more than 20% of mitochondrial counts). We extracted only the *fgfr3*+/+ control group, called WT in this article, because it had no genetic modification, and obtained an expression matrix with 21 176 genes and 9509 cells. The matrix dimensions were reduced by running the 20 significant principal components against scaled data; a clustering resolution of 0.2 was used to refine the clusters. Data were visualized using the RunUMAP function that computed a new matrix of two-dimensional coordinates for each cell based on the distance between clusters. Next, we performed reclustering and a UMAP analysis of cluster 4 (OB = osteoblasts). A clustering resolution of 0.2 was used to discriminate between immature and mature osteoblasts.

### CRISPR-Cas13d-mediated knockdown of *sgms2a* and *sgms2b*

CRISPR-Cas13d mediated knockdown of *sgms2a* and *sgms2b* was performed according to the protocol by Kushawah et al.^15^ Both *sgms2a* and *sgms2b* display several different transcript variants (Table S1). The most conserved and the longest transcripts coding for the main functional isoforms of *sgms2a* and *sgms2b* were selected for Cas13d gRNA design (*sgms2a-203*: ENSDART00000141476.4 and *sgms2b-201* (ENSDART00000053571.6)) (Table S1 and Figure S2 and S3). Briefly, the RNA secondary structures of zebrafish *sgms2a* and *sgms2b* coding regions were analyzed using RNAfold software (http://rna.tbi.univie.ac.at//cgi-bin/RNAWebSuite/RNAfold.cgi).^29^ The Cas13d gRNA sequences, targeting the predicted secondary structures, were designed using cas13design software (https://cas13design.nygenome.org).^30^^,^^31^ Three Cas13d gRNAs were utilized for both *sgms2a* and *sgms2b* (Table 1 and Figure S2 and S3). To ensure the design of the gRNAs specifically and uniquely target the *sgms2a* and *sgms2b* coding sequences in silico, the selected gRNAs were confirmed no significant off-target matches using BLAST^32^ against the zebrafish RefSeq. The pT3TS-RfxCas13d (Addgene) was linearized with XbaI (New England Biolabs). The linearized plasmid was used as a template for Cas13d mRNA synthesis using the T3 Message Machine kit (Ambion, Thermo Fisher). Cas13d mRNA was purified using the RNeasy Mini kit (Qiagen). The gRNAs were synthesized using the cloning-free PCR method. 2 nL mixture containing 200 pg of cas13d mRNA and 450 pg of multiple gRNAs targeting *sgms2a* or *sgms2b* (three gRNAs, 1:1:1 molecular ratio) or both (50:50 mixture of *sgms2a* and *sgms2b* targeting gRNAs) was injected into one-cell stage embryos. The injected control was cas13d mRNA without gRNAs (sham). CRISPR-Cas13d-transcriptome edited fish were generated in the WT AB zebrafish strain. Two independent CRISPR-Cas13d-knockdown experiments were performed. In the first experiment, 24 fish were injected in each group (*sgms2a* kd, *sgms2b* kd, *sgms2a+b* kd). In the second experiment, 25 fish were injected in each group (sham control, *sgms2a* kd, *sgms2b* kd, *sgms2a+b* kd). In addition, a similar number of uninjected WT controls were included in both experiments. Data from two independent experiments were combined, resulting in a total number of 49 WT, 25 sham, 49 *sgms2a* kd, 49 *sgms2b*, and 49 *sgms2a+b* kd zebrafish at day 0.

CRISPR-Cas13d-knockdown zebrafish embryo phenotype (normal, mild deformity, severe deformity, and dead) was evaluated at 2 dpf. The zebrafish were maintained on standard growth conditions (at 28.5 °C, 14 h light/10 h dark day-night cycle, fed from day 5 onwards) with a density of 24-25 fish per Petri dish (100 mm × 20 mm) in E3 embryo medium until day 7 when they were euthanized for cartilage and skeletal staining. The number of live fish was determined at 2 and 7 dpf to monitor fish survival using Leica DMi1 microscope attached to MC120 HD camera (Leica Microsystems).

### CRISPR-Cas13d-mediated *sgms2a* and *sgms2b* knockdown efficiency evaluation

The efficiency of CRISPR-Cas13d-mediated knockdown of *sgms2a* and *sgms2b* was evaluated on mRNA level by RT-qPCR at time points 3.5 h post-fertilization and 2, 5, and 7 dpf. For each time point, an independent set of embryos was injected (*n* = 25/group) (sham, *sgms2a* kd, *sgms2b* kd, and *sgms2a+b* kd) and raised until the desired age. The fish sacrification, RNA extraction, and RT-qPCR procedures were performed as described above.

### Cartilage and skeletal staining and image acquisition of 7 dpf CRISPR-Cas13d-knockdown larvae

CRISPR-Cas13d-kd and WT zebrafish were sacrificed at 7 dpf and fixed for 2 h in 4% PFA. Zebrafish cartilage and skeletal elements were stained with Alcian Blue 8 GX (C.I. 74240, Sigma-Aldrigh) and Alizarin Red S (C.I. 58005, Sigma-Aldrigh) stains, respectively. A two-color acid-free double staining protocol by Walker and Kimmel was followed by using 0.02% Alcian Blue, 0.005% Alizarin Red S, and 40 mM MgCl_2_.^33^ Zebrafish were mounted on 3% methylcellulose (M-0387, Sigma-Aldrigh) and imaged with Leica microscopes (DMi1 equipped with MC120 HD camera/DM2500 LED equipped with Flexacam C5 camera/Ivesta 3 with integrated camera) (Leica Microsystems).

### Morphological evaluation of 7 dpf cartilage and skeletal-stained CRISPR-Cas13d-knockdown larvae

Parameters of the 7 dpf Alizarin Red S and Alcian Blue-stained CRISPR-Cas13d-kd zebrafish larvae were measured from microscope images using the public domain ImageJ software.^34^ The following parameters were measured: standard length, notochord bending angle, head length, Meckel’s cartilage angle, ceratohyal cartilage angle, palatoquadrate-Meckel’s cartilage angle, palatoquadrate-ceratohyal angle, eye width, interocular distance, and parasphenoid bone area. In addition, notochord phenotype (eight categories) and mineralization (normal, decreased, and no mineralization), craniofacial phenotype (normal, mild deformity, and severe deformity), and eye phenotype (normal, cyclopia, hypotelorism, and uneven size) were determined for the larvae. Evaluation of the larvae morphology was done by at least two independent observers.

### Statistical analyses

Data analysis utilized GraphPad Prism version 9.4.1 software for Windows (GraphPad Software). For the RT-qPCR data, unpaired *t*-tests and ordinary one-way ANOVA followed by Dunnett’s test were utilized. The parameters of the stained zebrafish were analyzed as follows. First, the normality of the data was assessed using a Q-Q plot and Shapiro-Wilk’s test. *p*-values were generated by a nonparametric Kruskal–Wallis followed by Dunn’s test or parametric one-way ANOVA followed by Dunnett’s test. For categorical variables, Fisher’s exact test and Bonferroni correction were utilized (IBM SPSS Statistics for Windows, Version 29.0.2.0). The number of the analyzed fish is indicated in the figures. *p*-value <.05 was considered statistically significant.

### Protein sequence comparison

The similarity between human SMS2 and zebrafish Sms2a and Sms2b protein sequences were compared in the UniProt Knowledgebase^35^ using the Clustal Omega for multiple sequence alignment^36^ and Clustal 2.1 for percent identity matrix. The following UniProt sequences were used: SMS2: Q8NHU3; Sms2a: A0A8M1NT99; and Sms2b: Q6DEI3.

## Results

### Human *SGMS2* gene orthologs in zebrafish

**Figure 1:**
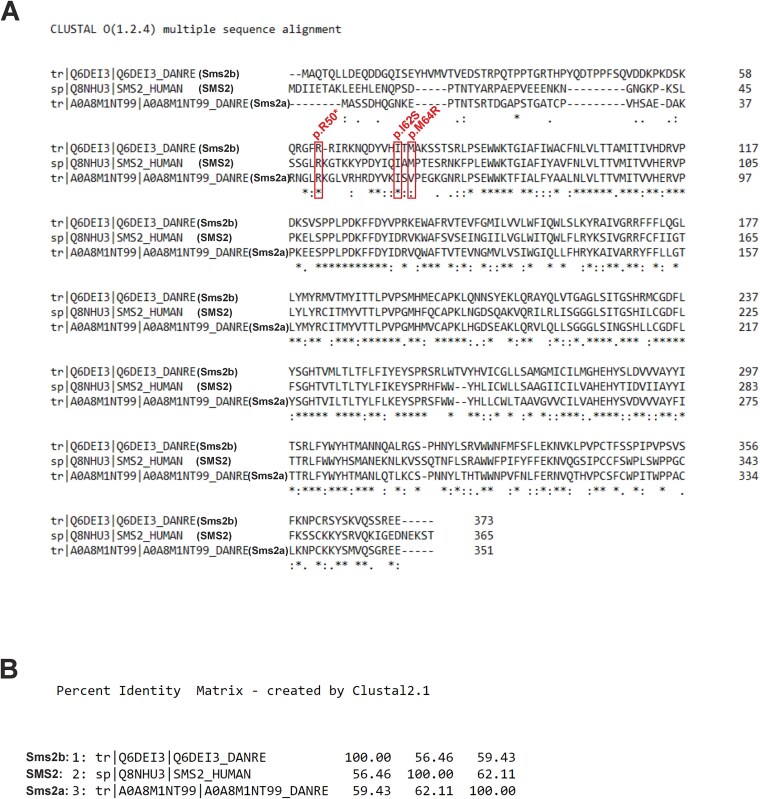
Protein sequence comparison of SMS2, Sms2a, and Sms2b. (A) Multiple sequence alignment of human SMS2 (365 aa) and zebrafish Sms2a (351 aa) and Sms2b (373 aa). Residues are identical at 165 positions (marked as stars). Positions of three human SMS2 pathogenic variants (p.R50*, p.I62S, p.M64R)^4^ have been indicated with boxes. Human SMS2 residues R50 and I62 are detected in both Sms2a and Sms2b sequences. Human SMS2 residue M64 is detected only in Sms2b sequence. “*” means that the residues in that column are identical in all sequences in the alignment; “**:**” means that conserved substitutions have been observed, that is, amino acid is replaced by one having similar characteristics; “**.**” means that semi-conserved substitutions are observed, that is, amino acids having similar shape. (B) Protein sequence percent identity matrix. Sms2a and Sms2b share 59% sequence identity. Sms2a and SMS2 share 62% sequence identity. Sms2b and SMS2 share 56% sequence identity. The following UniProt sequences were used: SMS2, Q8NHU3; Sms2a, A0A8M1NT99; Sms2b, Q6DEI3. The protein sequences were compared in the UniProt Knowledgebase^35^ using the Clustal Omega for multiple sequence alignment^36^ and Clustal 2.1 for percent identity matrix.

Human *SGMS2* gene encodes the protein SMS2 of 365 amino acids. The zebrafish genome includes two orthologs of human *SGMS2*, *sgms2a and sgms2b*, encoding proteins of 351 (Sms2a) and 373 (Sms2b) amino acids. Protein sequence comparison of SMS2, Sms2a, and Sms2b showed identical residues at 165 positions (Figure 1A). Human SMS2 N-terminal residues R50 and I62 harboring pathogenic variants in the affected CDL patients were identical in both Sms2a and Sms2b and residue M64 was identical only in Sms2b (Figure 1A). Protein sequence identity was 62% between Sms2a and SMS2, 56% between Sms2b and SMS2, and 59% between Sms2a and Sms2b (Figure 1B).

### *sgms2a* and *sgms2b* mRNA expression in WT zebrafish embryos and early larvae by whole-mount in situ hybridization and RT-qPCR

**Figure 2:**
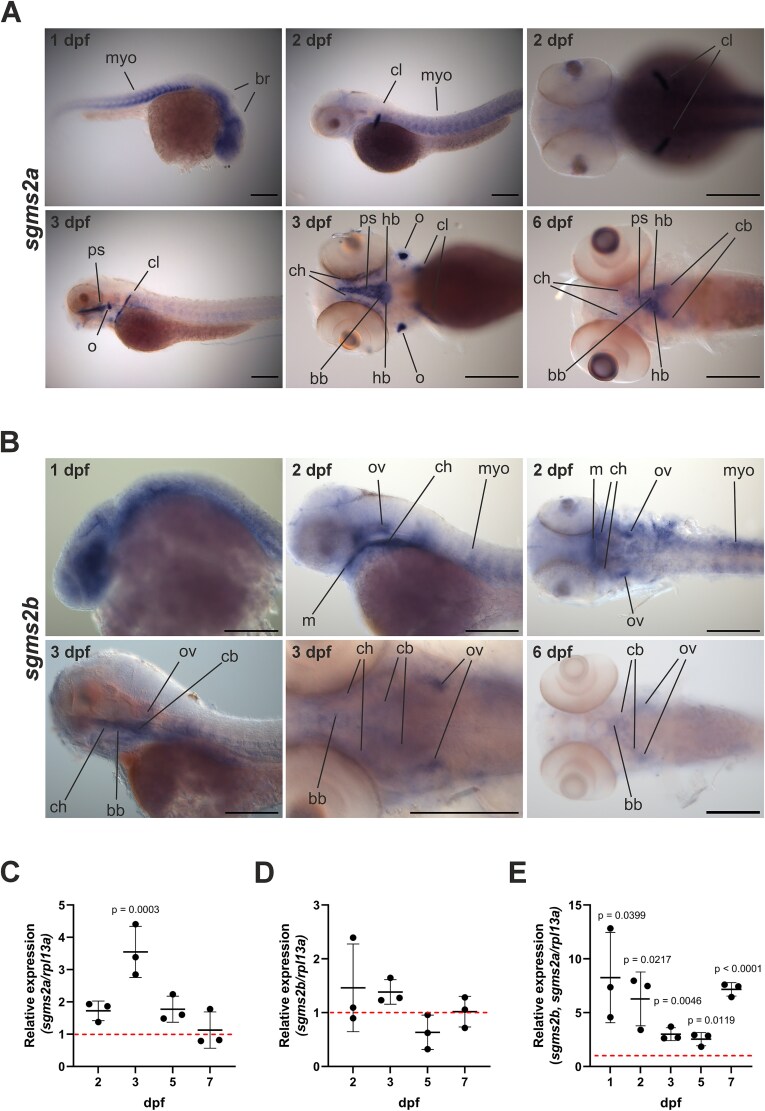
*sgms2a* and *sgms2b* mRNA expression in WT zebrafish embryos and early larvae by whole-mount in situ hybridization and RT-qPCR. (A) Spatial expression pattern of *sgms2a* mRNA at 1, 2, 3, and 6 dpf WT zebrafish by whole-mount in situ hybridization. Expression-specific structures are indicated with abbreviations. *sgms2a* negative control is presented in Figure S1. (B) Spatial expression pattern of *sgms2b* mRNA at 1, 2, 3, and 6 dpf WT zebrafish by whole-mount in situ hybridization. Expression-specific structures are indicated with abbreviations. *sgms2b* negative control is presented in Figure S1. (C-E) Expression patterns of *sgms2a* and *sgms2b* (relative expression to *rpl13a*) at 1, 2, 3, 5, and 7 dpf WT whole-body zebrafish by RT-qPCR. In (C) and (D), expression at each time point was compared to the expression at time point 1 dpf (expression at 1 dpf was set to 1 (shown as dotted line)). In (E), *sgms2b* and *sgms2a* expression was compared (the expression of *sgms2a* at each time point was set to 1 (shown as dotted line). Data are shown as scatter dot plots with mean ± SD. The *p*-values were determined using an unpaired *t*-test. The results from three independent experiments were combined. *p* < .05 was considered statistically significant. Only significant *p*-values were presented. bb, basibranchial; br, brain; cb, ceratobrancial; ch, ceratohyal; cl, cleithrum; dpf, days post-fertilization; hb, hypobranchial; m, Meckel’s cartilage; myo, myotome; o, opercle; ov, otic vesicle; ps, parasphenoid. The whole-mount in situ hybridization experiments were performed twice (*n* = 5-10 embryos or larvae/time point/experiment). Zebrafish orientation in both (A) and (B): upper row from left to right: lateral, lateral, dorsal; lower row from left to right: lateral, dorsal, dorsal. Scale bar = 200 μm.

We first visualized the spatial expression patterns of both *sgms2a* and *sgms2b* mRNA in WT zebrafish at 1, 2, 3, and 6 dpf by WISH (Figures 2A and B and Figure S1). *sgms2a* was highly expressed in the head region, including the brain and the myotome at 1 dpf and in the craniofacial skeletal structure cleithrum and in the myotome at 2 dpf (Figure 2A). At 3 dpf, *sgms2a* expression was concrete for the craniofacial skeletal elements: triangular parasphenoid bone, bilateral dot-shaped opercle, and bilateral cleithrum as well as ceratohyal cartilage and the oval-shaped region near the posterior part of the parasphenoid bone corresponding basibranchial and hypobranchial cartilage structures (Figure 2A). At 6 dpf, *sgms2a* expression was detected in the posterior part of the parasphenoid bone, basibranchial and hypobranchial cartilage, as well as at low level in ceratohyal cartilage and in ceratobranchial pairs (Figure 2A). *sgms2b* expression was ubiquitously detected in the head and the trunk at 1 dpf and more specifically localized in the pharyngeal arch cartilage structures (Meckel’s cartilage and ceratohyal), otic vesicles, and myotome at 2 dpf (Figure 2B). At 3 dpf, *sgms2b* expressed in the ceratohyal, ceratobranchial pairs, and basibranchial, as well as in otic vesicles (Figure 2B). At 6 dpf, *sgms2b* expression was detected, although the expression levels were very low, in the basibranchial, ceratobranchial pairs, and otic vesicles (Figure 2B).

**Figure 3:**
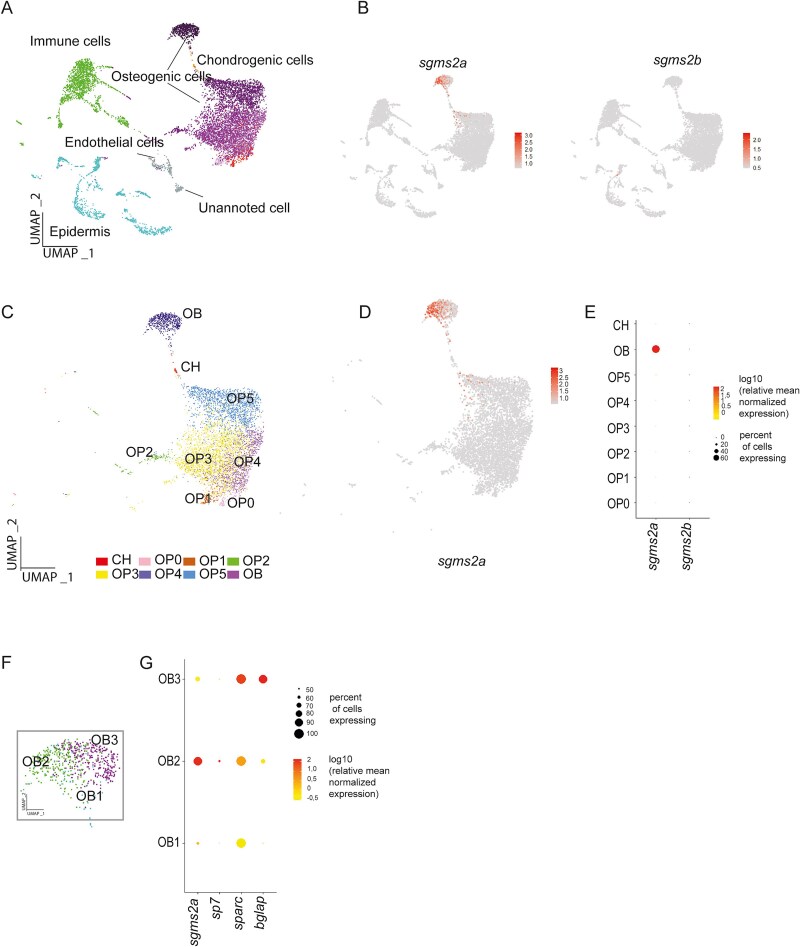
mRNA expression of *sgms2a* and *sgms2b* in osteogenic cell subpopulations during cranial vault development in WT juvenile zebrafish by single-cell RNA sequencing. (A) UMAP analysis of cranial vault pooled cells from WT zebrafish and (*n* = 9509), with the cell type assignments for clusters. (B) The feature plots for *sgms2a* and *sgms2b*. The color intensity corresponds to the mean normalized expression level. (C) UMAP pictures representing osteogenic (OC) cells (including osteoprogenitors (OP0 to OPB5) and osteoblasts (OB)) and chondrogenic (CH) clusters (*n* = 6096). (D) The feature plot for *sgms2a* in OC and CH cells. *sgms2b* expression was not detected. The color intensity corresponds to the mean normalized expression level. (E) A dot plot showing *sgms2a* and *sgms2b* mRNA expression in OC and CH clusters. The dot diameter corresponds to the fraction of cells expressing each gene in each cluster, and the scale color corresponds to the mean normalized expression level. (F) UMAP analysis of pooled cells from WT zebrafish from the OB cluster (*n* = 605). (G) A dot plot showing *sgms2a*, *sp7*, *sparc*, and *bglap* mRNA expression in OB clusters. The dot diameter corresponds to the proportion of cells expressing each gene in each cluster, as shown on the scale. The color corresponds to the mean normalized expression level.

Second, we studied expression patterns of *sgms2a* and *sgms2b* by RT-qPCR using WT whole-body embryos and larvae in the early days of development. The expression of *sgms2a* increased in WT larvae at 2, 3, 5, and 7 dpf compared with the expression at 1 dpf (Figure 2C). The highest expression was observed at 3 dpf (3.5-fold, *p* = .0003) (Figure 2C). In contrast, the expression of *sgms2b* in WT larvae showed a more stable pattern at 2, 3, 5, and 7 dpf compared with at 1 dpf (Figure 3D). When comparing the expression levels of *sgms2b* with *sgms2a* at 1, 2, 3, 5, and 7 dpf, *sgms2b* levels were significantly higher throughout (Figure 3E).

### *sgms2a* and *sgms2b* mRNA expression in WT juvenile zebrafish calvaria cells by single-cell RNA sequencing

We further evaluated the expression of *sgms2a* and *sgms2b* mRNA in calvaria cells in WT juvenile zebrafish. We utilized a previously reported dataset by Dambroise et al.^27^ of cranial vault samples analyzed by single-cell RNA sequencing. By extracting the WT data from the dataset and studying different cell clusters, *sgms2a* expression was high in osteogenic cells whereas *sgms2b* expression was not detected in this cell type (Figure 3A and B). More precisely, *sgms2a* was mainly expressed in immature osteoblast cells and slightly expressed in osteoprogenitor cells and mature osteoblasts during calvaria development (Figure 3C-G). Alongside *sgms2a*, osteogenic markers *sp7*, *sparc*, and *bglap* expressed in osteoblast clusters (Figure 3G).

### CRISPR-Cas13d-mediated knockdown of *sgms2a* and *sgms2b*

**Figure 4:**
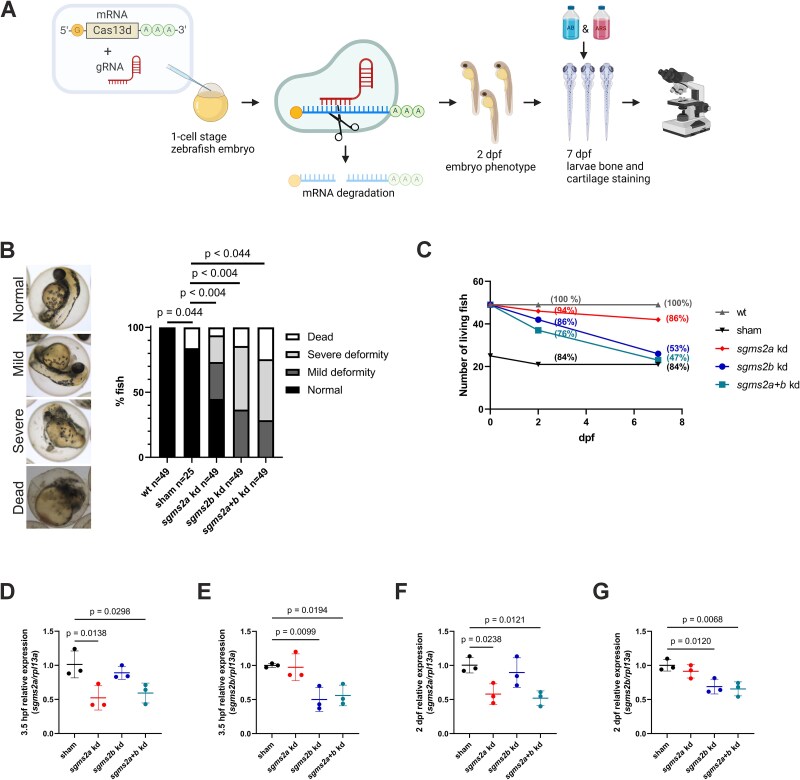
CRISPR-Cas13d-mediated knockdown of *sgms2a* and *sgms2b.* (A) Schematic illustration of CRISPR-Cas13d-kd experimental design. (B) Embryo phenotype at 2 dpf. Embryos were classified into four categories: normal, mild deformity, severe deformity, and dead. Representative images of each category are presented on the left side of the graph. The WT, the *sgms2a* kd, the *sgms2b* kd, and the *sgms2a+b* kd embryos were compared with the sham embryos (Fisher’s exact test and Bonferroni correction). (C) Number of living fish during the 7-d experiment. The percentage of living fish at 2 and 7 dpf (the number of fish at 2 or 7 dpf compared with the original number of the injected embryos at day 0) for each group has been indicated in parenthesis. At day 7, larvae were euthanized for bone and cartilage staining. (D-G) Expression of *sgms2a* and *sgms2b* mRNA (relative expression to *rpl13a*) at 3.5 h post fertilization (hpf) and 2 dpf in sham, *sgms2a* kd, *sgms2b* kd, and *sgms2a+b* kd embryos by RT-qPCR. Data are shown as scatter dot plots with mean ± SD. The results from three independent experiments were combined. The *sgms2a* kd, the *sgms2b* kd, and the *sgms2a+b* kd embryos were compared with the sham embryos (ordinary one-way ANOVA for multiple comparisons). *p* < .05 was considered statistically significant. Only significant *p*-values were presented.

A schematic illustration of CRISPR-Cas13d-knockdown experimental design is presented in Figure 4A. The *sgms2a* and *sgms2b* transcripts were targeted separately and together resulting in three different kd groups (*sgms2a* kd, *sgms2b* kd, and *sgms2a+b* kd). In addition, an injection control (sham) was generated. Phenotypes of the kd, the sham, and the WT embryos were evaluated at 2 dpf. Increased number of embryos presented other than normal phenotype (mild/severe deformity or dead) in the kd groups (*p* < .004) compared with the sham control group (Figure 4B). Of note, the number of dead embryos increased in the sham group compared to the WT group (*p* = .044) (Figure 4B). We followed the fish survival during the 7-d experiment. The number of living fish decreased remarkably in the *sgms2b* kd and *sgms2a+b* kd groups: at day 7, 53% of the *sgms2b* kd larvae and 47% of the *sgms2a+b* kd larvae were alive from the original number of the injected embryos at day 0 (Figure 4C).

CRISPR-Cas13d-mediated knockdown efficiency was evaluated at mRNA level by RT-qPCR. At 3.5 h post-fertilization (hpf), when transcripts are already considered to originate from zygote, *sgms2a* expression levels were significantly decreased in the *sgms2a* and *sgms2a+b* kd groups (0.52-fold (*p* = .0138) and 0.59-fold (*p* = .0298)), respectively, but not in the *sgms2b* kd group, compared with the sham group (Figure 4D). Similarly at 3.5 hpf, *sgms2b* expression levels were significantly decreased in the *sgms2b* and *sgms2a+b* kd groups (0.50-fold (*p* = .0099) and 0.56-fold (*p* = .0194)), respectively, but not in the *sgms2a* kd group, compared with the sham group (Figure 4E). At 2 dpf, when transcripts are considered as primarily of zygotic origin, *sgms2a* expression levels were significantly decreased in the *sgms2a* and *sgms2a+b* kd groups (0.58-fold (*p* = .0238) and 0.52-fold (*p* = .0121)), respectively, but not in the *sgms2b* kd group, compared with the sham group (Figure 4F). Similarly at 2 dpf, *sgms2b* expression levels were significantly decreased in the *sgms2b* and *sgms2a+b* kd groups (0.69-fold (*p* = .0120) and (0.65-fold (*p* = .0068)), respectively, but not in the *sgms2a* kd group, compared with the sham group (Figure 4G). We further evaluated the *sgms2a* and *sgms2b* expression levels at later time points, that is, 5 and 7 dpf, kd, but no differences were observed between the sham and the kd groups (Figure S4). These results confirm temporary knockdown of target genes.

### Notochord features in 7 dpf *sgms2a*, *sgms2b*, and *sgms2a+b* knockdown zebrafish larvae

**Figure 5:**
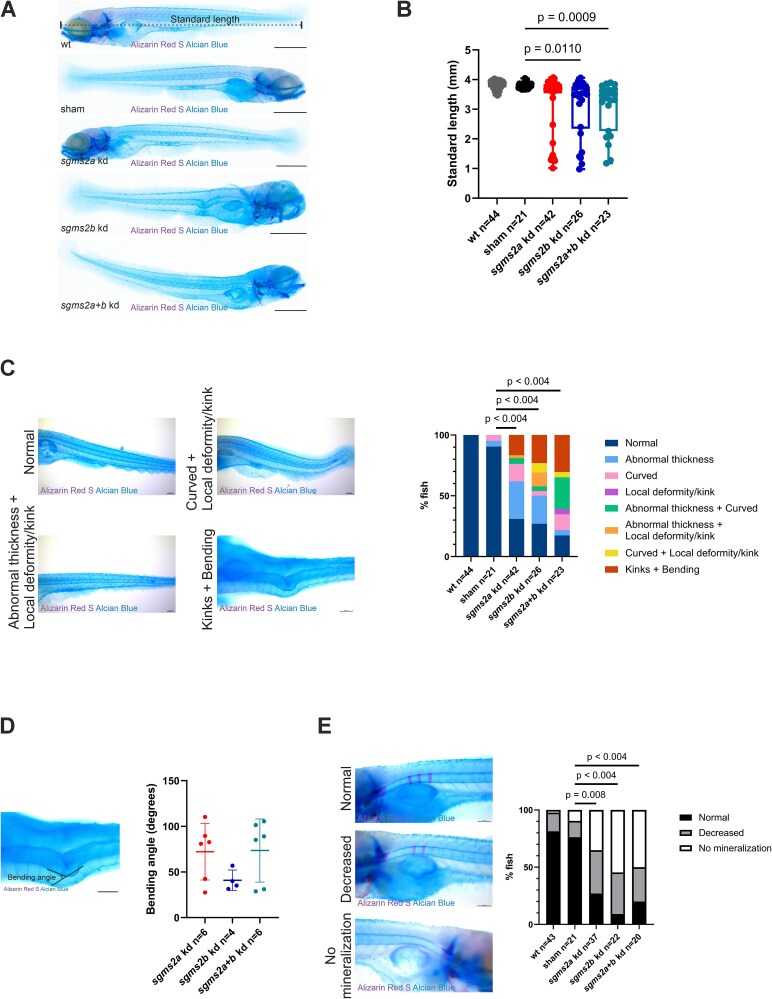
Notochord features in 7 dpf Alizarin Red S and Alcian Blue-stained *sgms2a* kd, *sgms2b* kd, and *sgms2a+b* kd larvae. (A) Representative lateral view whole-body images of Alizarin Red S and Alcian Blue-stained 7 dpf WT, sham, *sgms2a* kd, *sgms2b* kd, and *sgms2a+b* kd zebrafish larvae. Measurement of standard length is illustrated in the WT zebrafish. (B) Standard length. Data are shown as a box plot. The *p*-values were generated by ordinary one-way ANOVA for multiple comparisons (sham larvae vs WT, *sgms2a* kd, *sgms2b* kd and *sgms2a+b* kd larvae). (C) Notochord phenotype. Data are shown as a bar chart. The *p*-values were generated by Fisher’s exact test and Bonferroni correction (sham larvae vs WT, *sgms2a* kd, *sgms2b* kd and *sgms2a+b* kd larvae). The notochord phenotypes were classified into eight categories: normal, abnormal thickness, curved, local deformity/kink, abnormal thickness + curved, abnormal thickness + local deformity/kink, curved + local deformity, and kinks + bending. Representative images of the following notochord features: normal, abnormal thickness + local deformity/kink, curved + local deformity/kink, and kinks and bending, are presented on the left side of the bar chart. (D) Bending angle. Data are shown as a scatter dot plot with mean ± SD. The bending angles between the *sgms2a* kd, the *sgms2b* kd, and the *sgms2a+b* kd larvae were compared by ordinary one-way ANOVA for multiple comparisons. (E) Early vertebral element (notochord sheath) mineralization. Mineralization was classified into three categories: normal (at least 3 mineralized elements), decreased (less than 3 mineralized elements), and no mineralization (0 mineralized elements). The representative images of the categories are presented on the left side of the bar chart. Data are shown as a bar chart. The *p*-values were generated by Fisher’s exact test and Bonferroni correction (sham larvae vs WT, *sgms2a* kd, *sgms2b* kd and *sgms2a+b* kd larvae). Abbreviation: kd, knockdown. Scale bar = 500 μm (A) and 100 μm (C-E). *p* < .05 was considered statistically significant. Only significant *p*-values were presented.

CRISPR-Cas13d-kd zebrafish larvae were euthanized at 7 dpf, stained with Alizarin Red S and Alcian Blue, and imaged to evaluate morphological features. Whole-body images of the WT, sham, *sgms2a* kd, *sgms2b* kd, and *sgms2a+b* kd larvae are presented in Figure 5A. The *sgms2a* kd, *sgms2b* kd, and *sgms2a+b* kd larvae showed abnormalities in the notochord and craniofacial structures (Figure 5A). A significant decrease in standard length was observed in the *sgms2b* kd and the *sgms2a+b* kd larvae compared with the sham control larvae (*p* = .011 and *p* = .0009, respectively) (Figure 5B). Notochord abnormalities were increased in all kd larvae (*p* < .004) compared with the sham control larvae (Figure 5C). Notochord abnormalities were classified into eight categories: normal, abnormal thickness, curved, local deformity/kink, abnormal thickness + curved, abnormal thickness + local deformity/kink, curved + local deformity, and kinks + bending. One of the most striking notochord abnormalities was kinks + bending of the notochord axis. We further measured the notochord bending angle in the *sgms2a* kd (*n* = 6), *sgms2b* kd (*n* = 4), and *sgms2a+b* kd (*n* = 6) larvae (Figure 5D). Larger bending angles were observed in the *sgms2a* kd and the *sgms2a+b* kd larvae but no significant differences were reached between the kd groups (Figure 5D). Moreover, we observed differences in Alizarin Red S-staining (indicating mineralization) of the notochord. All kd larvae showed significantly decreased notochord mineralization (*sgms2a* kd larvae: *p* = .008; *sgms2b* and *sgms2a+b* kd larvae: *p* < .004) compared with the sham control larvae (Figure 5E).

### Craniofacial and eye defects in 7 dpf *sgms2a*, *sgms2b*, and *sgms2a+b* knockdown zebrafish larvae

**Figure 6:**
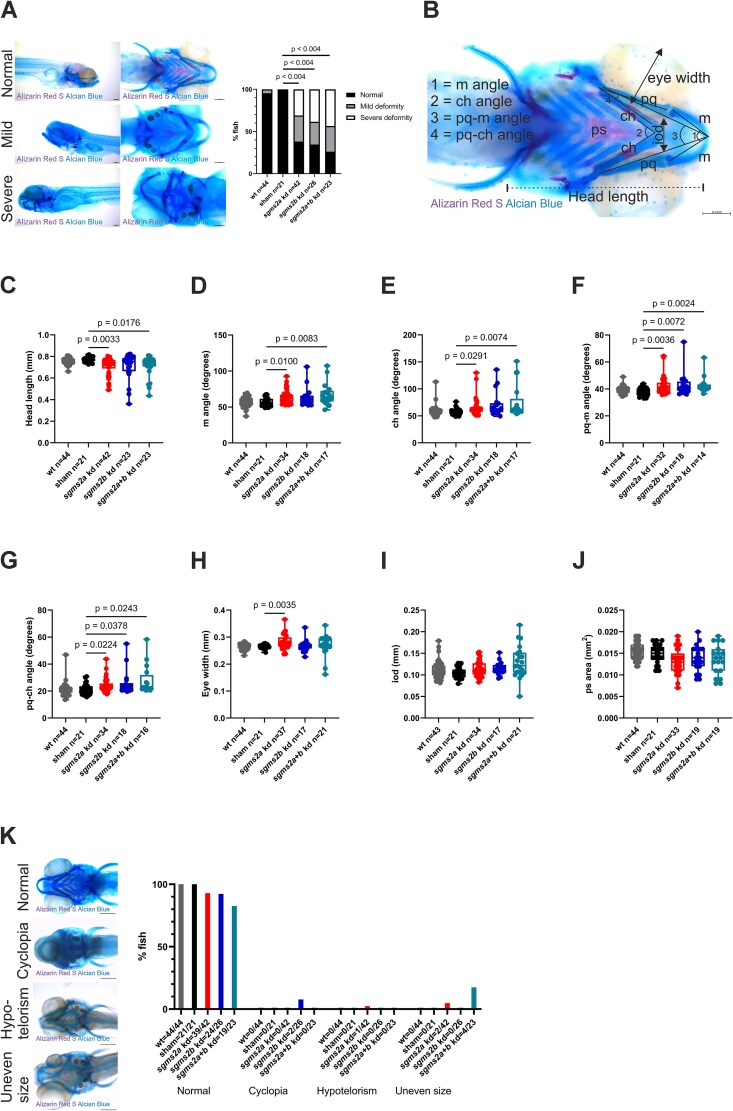
Craniofacial and eye features in 7 dpf Alizarin Red S and Alcian Blue-stained *sgms2a* kd, *sgms2b* kd, and *sgms2a+b* kd larvae. (A) Craniofacial deformity. Craniofacial phenotype was classified into three categories: normal, mild deformity, and severe deformity. Representative images of each category are presented on the left side of the graph (left row: lateral view, right row: ventral view). Cartilage elements are stained in Alcian Blue and mineralized skeletal elements in Alizarin Red S. Data are shown as a bar chart. The *p*-values were generated by Fisher’s exact test and Bonferroni correction (sham larvae vs WT, *sgms2a* kd, *sgms2b* kd, and *sgms2a+b* kd larvae). (B) Representative ventral view image of Alizarin Red S and Alcian Blue-stained zebrafish larvae head region with measured parameters indicated. Measurements of parameters are presented in box plots (C-J). (C) Head length. (D) M angle. (E) Ch angle. (F) Pq-m angle. (G) Pq-ch angle. (H) Eye width. (I) IOD. (J) PS area. In (C-J), the *p*-values were generated by ordinary one-way ANOVA for multiple comparisons (sham larvae vs WT, *sgms2a* kd, *sgms2b* kd, and *sgms2a+b* kd larvae). (K) Eye deformity. Data are shown as a bar chart. Eye phenotype was classified into four categories: normal, cyclopia, hypotelorism, and uneven size. The number of zebrafish larvae with the phenotype are indicated in the x-axis. Representative images of each category are presented on the left side of the bar chart. ch, ceratohyal; iod, interocular distance; kd, knockdown; m, Meckel’s cartilage; pq, palatoquadrate; ps, parasphenoid bone. Scale bar = 100 μm (A and B), and 200 μm (K). *p* < .05 was considered statistically significant. Only significant *p*-values were presented.

The craniofacial deformity was increased in all kd larvae compared to sham control larvae (*p* < .004) (Figure 6A). The craniofacial elements were further measured using the following parameters: head length, Meckel’s (m) angle, ceratohyal (ch) angle, palatoquadrate-Meckel’s (pq-m) angle, palatoquadrate-ceratohyal (pq-ch) angle, eye width, interocular distance (iod), and parasphenoid (ps) bone area (Figure 6B). A significant decrease in head length was observed in the *sgms2a* kd and *sgms2a+b* kd larvae (*p* = .0033 and *p* = .0176, respectively) but not in the *sgms2b* kd larvae compared with the sham control larvae (Figure 6C). The results showed significantly increased Meckel’s and ceratohyal angles in the *sgms2a* kd and *sgms2a+b* kd larvae but not in the *sgms2b* kd larvae compared with the sham control larvae (Figures 6D and E). Also, palatoquadrate-Meckel’s and palatoquadrate-ceratohyal angle were significantly increased in all kd larvae compared with the sham control larvae (Figures 6F and G). The eye width was also significantly increased only in the *sgms2a* kd larvae compared with the sham control larvae (*p* = .0035) (Figure 6H). Lastly, interocular distance tended to be increased in all kd larvae, but no significant difference was reached compared with the sham control larvae (Figure 6I). The difference in Alizarin Red S-stained parasphenoid bone area was not significant between the kd larvae and the sham control larvae (Figure 6J).

Furthermore, eye abnormalities, including cyclopia, hypotelorism, and uneven-sized eyes, were observed in the kd larvae but not in the sham or the WT control larvae (Figure 6K). Altogether, cyclopia was observed in 2/26 of the *sgms2b* kd larvae, hypotelorism was observed in 1/42 of the *sgms2a* kd larvae, and uneven sized eyes (left eye-right eye width ratio >1.20 or <0.90) in 2/42 of the *sgms2a* kd and 4/23 of the *sgms2a+b* kd larvae (Figure 6K).

## Discussion

Previous clinical and functional studies have shown *SGMS2* to play a critical role in bone metabolism and defects in its function to result in severe skeletal pathologies. Many of the molecular details underlying sphingolipid metabolism-related skeletal disorders, however, remain unknown. In this study, we analyzed the functional role of human *SGMS2* gene orthologs *sgms2a* and *sgms2b* in zebrafish. We investigated *sgms2a* and *sgms2b* mRNA expression in WT zebrafish during the first week of development by WISH and RT-qPCR, and in month-old juvenile WT zebrafish calvarial cells by single-cell RNA sequencing. We also utilized a novel transcriptome editing tool, CRISPR-Cas13d, and developed *sgms2a*, *sgms2b*, and *sgms2a+b* kd zebrafish models to study in detail embryo phenotype at 2 dpf and bone and cartilage tissue characteristics and morphological features at 7 dpf zebrafish. The results showed that *sgms2a* and *sgms2b* were expressed in WT zebrafish in myotome and craniofacial skeleton during the first week of development and *sgms2a* in calvarial osteogenic cells during the juvenile stage. We also observed that CRISPR-Cas13d-knockdown of *sgms2a* and *sgms2b* had detrimental effects on embryonic development and compromised notochord and craniofacial development in zebrafish larvae. These observations bring new insight into the role of *SGMS2* in the musculoskeletal system and report the utility of CRISPR-Cas13d-tool in skeletal research to obtain preliminary insights into the gene’s functions and to provide clues for molecular mechanism driving pathological conditions.

The proteins encoded by *sgms2a* and *sgms2b* shared approximately 60% sequence identity with each other and with human SMS2, indicating that they are likely to exhibit functional similarities but also differences. Human SMS2 residues, R50, I62, and M64, substituted by OP-associated variants,^4^ were detected in zebrafish orthologues: R50 and I62 residues were detected in both Sms2a and Sms2b and M64 was detected only in Sms2b. Both zebrafish *sgms2* genes were considered relevant for further studies.

During the first week of development, WISH analyses revealed *sgms2a* and *sgms2b* mRNA expression in the musculoskeletal system in WT zebrafish, with both genes expressed in the myotome and the craniofacial cartilage, suggesting their central roles in cartilage and muscle tissue development. Additionally, *sgms2a* expression was detected in craniofacial skeletal elements at 3 dpf, thus indicating a pivotal role for the gene in bone tissue formation during early development. Interestingly, single-cell RNA sequencing of WT 1-mo-old juvenile zebrafish calvaria cells demonstrated high *sgms2a* expression together with other osteogenic markers (*sp7*, *sparc*, and *bglap*) in osteogenic cells. *sgms2a* was particularly expressed in immature osteoblasts, underscoring the role of *sgms2a* in bone formation also at the juvenile stage. In contrast, the expression of *sgms2b* expression was not detected in WT juvenile zebrafish calvarial osteogenic or chondrogenic cells, indicating its role primarily pertain to cartilage tissue during early development. Notably, *sgms2b* was also expressed in the otic vesicles during 1-6 dpf. The otic vesicle participates in the early development of hearing, giving rise to the inner ear’s main sensory components.^37^ Further efforts are needed to clarify the exact expression of *sgms2b* in different inner ear components. In addition, *sgms2a* expressed in the brain at 1 dpf, detected by WISH, implicating the gene in brain functions. Detailed analyses of zebrafish brain slice sections are required to explore *sgms2a* expression in specific brain regions. Importantly, human patients with pathogenic *SGMS2* variants, portray a variety of neurological manifestations.^6^

The expression profiles of *sgms2a* and *sgms2b*, determined also by RT-qPCR in WT whole-body zebrafish embryos and larvae during 1-7 dpf, were observed to align with the spatial expression profiles of both genes detected by WISH. The results also showed that *sgms2b* mRNA was more highly expressed than *sgms2a* mRNA implicating a more dominant role of *sgms2b* transcripts, at least during the early stages of zebrafish development. However, as mRNA expression levels are not always directly comparable to protein levels, the spatial expression of both *sgms2a* and *sgms2b* should also be confirmed at the protein level.

By utilizing the novel CRISPR-Cas13d-method, that has been highlighted as an efficient tool to interrogate embryonic gene function,^15^ we created three different kd zebrafish models: *sgms2a* kd, *sgms2b* kd, and *sgms2a+b* kd. To our knowledge, no previous studies utilizing CRISPR-Cas13d-tool to model skeletal phenotype, are available. A significant decrease in the *sgms2a* and *sgms2b* mRNA expression levels by CRISPR-Cas13d-knockdown were confirmed during the first 2 d after fertilization. The earliest embryonic time point to inspect *sgms2a* and *sgms2b* knockdown, 3.5 hpf, was selected based on the Expression Atlas data^38^ of *sgms2a* and *sgms2b* by choosing the timepoint that occurs after zygotic genome activation and at which both genes are expressed at detectable levels. At 3.5 hpf, approximately 50% decrease in the *sgms2a* and *sgms2b* transcript levels were detected in the *sgms2a* and *sgms2b* kd groups and a slightly milder decrease, 41% and 44%, respectively, in the double kd group. Likewise, at 2 dpf, when still significantly decreased levels of *sgms2a* and *sgms2b* existed, increased numbers of deformed embryos were detected in the kd groups, implicating that fully functional *sgms2a* and *sgms2b* are essential for zebrafish embryonic development stages including cleavage, blastula, gastrula, segmentation, and pharyngula. During these stages, key developmental events, such as cell division, cell differentiation, establishment of basic body plan, and formation of precursors to all major organs, occur. The importance of *sgms2a* and *sgms2b* in early embryonic development was further supported by the WISH findings showing that in WT embryos, both genes have intense expression throughout the embryo at 1 dpf. Towards the endpoint of the experiment, CRISPR-Cas13d-knockdown was lost during 2 and 5 dpf but abnormal phenotypic features in the notochord and craniofacial region remained or the number of living fish decreased in the kd groups. This implies that regain of *sgms2a* and *sgms2b* expression fails to maintain the genes’ original functions in differentiated body structures with detrimental consequences during hatching and early larval developmental stages.

In addition, a noteworthy observation related to CRISPR-Cas13d-tool itself is that injection of purely Cas13d mRNA induced reduced survival rates (21/25, 84%) of sham control embryos at 2 dpf independent of *sgms2a/sgms2b* knockdown implying possible mechanical damage of microinjection procedure to the oocytes or cellular response to Cas13d mRNA. However, the original number of sham control fish (*n* = 25) was only half of the number of fish in other groups (*n* = 49). By increasing the number of sham control fish would be advantageous to interpret the effect of injections on survival rate trend more reliably. Despite increased number of dead fish in the sham group, no embryo deformities were observed in this particular group, in contrary to the kd groups, for which *sgms2a* and/or *sgms2b* targeting gRNAs were co-injected together with Cas13d mRNA.

The notochord features observed in the 7 dpf *sgms2a* kd, the *sgms2b* kd, and the *sgms2a+b* kd larvae included shortened axis, curvature, abnormal thickness, local deformities, and bending and kinks. These notochord features align with the phenotypic features of human CDL patients with short stature, vertebral fractures, and scoliosis.^4^ The notochord is a central embryonic midline structure typical of all chordates and plays crucial structural and signaling roles during vertebrae development.^39^^,^^40^ In zebrafish, the notochord rudiments are detected during the gastrula period (90%-epiboly stage, 9 h post-fertilization) when the axis and neural plate start to form.^21^ The notochord is a rod-like structure composed of a core of cells containing large fluid-filled vacuoles enclosed by a sheath of epithelial-like cells surrounded by a collagen-rich extracellular matrix sheath.^41^ Loss of vacuole formation or integrity in zebrafish is known to lead shortened anterior-posterior axis and cause kinks in the spine axis.^42^ Considering the role of *SGMS2* in sphingolipid metabolism, it is possible that vacuoles, which are membrane-surrounded organelles, can suffer from altered lipid distribution in membranes due to defective SMS2, challenging vacuole integrity, resulting in notochord defects, as seen in this study. Further, collagen fibril orientation in the extracellular matrix also affects the axial fluid pressure.^42^ Intriguingly, defects in the extracellular sheath matrix components, such as different types of collagens, result in a malformed notochord in the early embryo and dysmorphic vertebrae at later stages.^43^^,^^44^ Since defects in collagen fibril organization have been reported in bone biopsies from patients with *SGMS2* pathogenic variants,^4^ analyses of extracellular sheath matrix components, particularly collagens, are warranted in zebrafish *sgms2* deficiency models.

Besides abnormal notochord shape, we observed notochord sheath mineralization defects in the 7 dpf *sgms2a* kd, *sgms2b* kd, and *sgms2a+b* kd larvae. Of note, bone mineralization defects are also a central finding in human patients with pathogenic *SGMS2* variants.^4^ The formation of vertebrae differs between human and zebrafish. The human vertebrae centrum is instructed by somite patterning and polarization, whereas in zebrafish the centrum patterning is instructed by the notochord.^39^^,^^40^ In humans, vertebral bodies have a cartilaginous anlage, which later either mineralizes or is replaced by bone.^45^ In contrast, zebrafish vertebral centra form in the absence of cartilage and through the mineralization of the notochord sheath (chordacentrum), which is then surrounded by somite-derived intramembranous bone.^45^ Notochord sheath mineralization defects observed in *sgms2a* and *sgms2b* kd larvae might be explained by disturbances in notochord sheath-guided mechanisms in segmentation, mineralization, or both. It is also possible that *sgms2a* kd, *sgms2b* kd, and *sgms2a+b* kd larvae suffer from overall developmental delay, contributing to notochord sheath mineralization events.

Along with the notochord defects, we observed a deformed craniofacial phenotype and decreased head length in 7 dpf *sgms2a* kd, *sgms2b* kd, and *sgms2b* kd zebrafish larvae. Zebrafish develop a functional craniofacial skeleton within 5 dpf; the first cartilage elements in the head region are seen as early as 2 dpf.^13^^,^^46^ The craniofacial skeleton primarily comprises cartilage elements, later replaced by bones and a few intramembranous bones, including opercles.^47^ Here, we observed abnormalities in cartilage element dimensions and patterning. The defected cartilage elements showed abnormal organization/accumulation of chondrocytes, thus highlighting the importance of this cell type in *sgms2a* and *sgms2b* deficiency. These observed craniofacial cartilage defects in kd larvae were in line with our WISH findings: in WT zebrafish, both *sgms2a* and *sgms2b* expressed in craniofacial cartilage elements. Primary chondrocytes in human patients with *SGMS2* variants have not yet been studied.^4^ Interestingly, a study by Steinberg et al.^48^ reported the expression of SGMS2 in primary chondrocytes of osteoarthritis patients, further supporting the need to inspect chondrocyte phenotype in patients with *SGMS2*-related bone pathologies.

Although *sgms2a* kd, *sgms2b* kd, and *sgms2a+b* kd zebrafish presented apparent defects in the notochord sheath mineralization, no such strong effect was observed in the craniofacial bone elements at 7 dpf larvae despite that *sgms2a*, for instance*,* was explicitly detected to express in the WT zebrafish craniofacial bone elements at early development days by WISH analyses. We observed only a slight decrease in Alizarin Red S-stained parasphenoid bone area in kd larvae, particularly in *sgms2a* kd larvae, but these findings were not significantly different from those of sham control larvae. No apparent differences were observed either in opercle bone or cleithrum mineralization in kd larvae compared to sham control larvae (data not shown). Since mineralization starts at this stage and the efficiency of knockdown reduces over time, it would be relevant to inspect the craniofacial mineralization further at later stages in the stable *sgms2a* and *sgms2b* deficient zebrafish models.

We also observed morphological ocular defects, including cyclopia, hypotelorism, and uneven eye size, in a small number of 7 dpf *sgms2a* kd*, sgms2b* kd, and *sgms2a+b* kd larvae. Further, we observed increased eye width in *sgms2a* kd larvae and a trend of increased interocular distance in all kd larvae compared to sham control larvae. These ocular findings warrant further confirmation in later experiments. Interestingly, human patients with the pathogenic *SGMS2* p.Arg50* variants have presented with eye defects such as congenital glaucoma.^4^^,^^49^

Lastly, we detected mRNA expression of both *sgms2a* and *sgms2b* in myotome in 1-2 dpf larvae, implicating them also in muscle tissue. This preliminary finding warrants further studies in zebrafish and humans. Thus far, very little is known about muscle function in patients with *SGMS2*-related bone disease. One study showed muscle function deficits in patients harboring the *SGMS2* p.Arg50* variant.^50^ However, due to the limited number of subjects and confounding factors, such as obesity, this requires confirmation.^50^

Also, one central aspect in the future experiments will be to study the levels of sphingolipids in the *sgms2*-deficient zebrafish model to confirm the enzymatic loss-of-function phenotype. Attractive approaches include mass-spectrometry-based sphingolipidomic analyses combined with metabolomics as well as more detailed analyses tracking specific sphingolipid synthesis and localization. Although, no sphingolipid analyses were performed for the current *sgms2a/sgms2b* knockdown model, we hypothesize that *sgms2a/sgms2b* knockdown would lead to altered levels of SM and ceramide and the imbalance of these central sphingolipids would ultimately negatively affect systemic sphingolipid homeostasis.

A limitation of this study was that the limited number of CRISPR-Cas13d-kd zebrafish per group. Despite this, we were able to replicate the phenotype in two independent experiments and see statistical differences between kd larvae and sham control larvae in parameters evaluating phenotypic features. Second, we studied mRNA expression of *sgms2a* and *sgms2b* in WT zebrafish larvae in a limited period (during the first week) and at the one-time point at the juvenile stage (1-mo-old). A study of additional time points and specific tissues would be advantageous in fully characterizing expression patterns of the genes during zebrafish lifespan. Also, protein level expression data of Sms2a and Sms2b in zebrafish is still lacking. Finally, limited information on skeletal mineralization can be obtained by studying 7 dpf zebrafish. Defects or delays in early notochord sheath mineralization in kd zebrafish were detected at this stage, but craniofacial skeleton mineralization events should be followed at later stages. Ongoing work is being conducted to study the expression of *sgms2a* and *sgms2b* in WT zebrafish from early embryonic stages until adulthood and to generate stable *sgms2a* and *sgms2b* KO zebrafish lines to analyze musculoskeletal and neurological phenotype during zebrafish lifespan.

In conclusion, we report novel findings of the functional role of human *SGMS2* orthologs *sgms2a* and *sgms2b* in zebrafish. Our findings indicate that both *sgms2a* and *sgms2b* are required for normal embryonic development as well as notochord and craniofacial development in zebrafish larvae. In the CRISPR-Cas13d-developed models, kd of *sgms2a*, *sgms2b*, or *sgms2a*+*b* affected embryonic development and resulted in larvae with shortened axis, defects in notochord shape and sheath mineralization, and malformations in craniofacial skeleton and eyes. These kd models can be utilized as a preliminary step preceding the stable KO model to explore gene functions in vivo at early development days. A deeper understanding of the mechanisms underlying sphingolipid-related bone pathologies, such as CDL, is encouraged to improve therapeutic options for patients with defects in the sphingolipid metabolism pathway genes.

## Supplementary Material

Supplementary_Material_290925

## Data Availability

Data are available from the corresponding author upon reasonable request.
