# Supplementary material for: Utilizing CRISPR-Cas13d-knockdown in zebrafish to study a rare monogenic bone fragility syndrome
Source: JBMR Plus. 2025 Oct 23;9(11):ziaf153. doi: 10.1093/jbmrpl/ziaf153 (PMC12548727; doi:10.1093/jbmrpl/ziaf153)
Supplement: Supplementary_Material_290925 [file supplementary_material_290925.docx]

**Supplementary Material**

**Utilizing CRISPR-Cas13d-knockdown in zebrafish to study a rare monogenic bone fragility syndrome**

Kirsi Määttä^*1, 2^, Yu-Chia Chen^*3, 4^, Sandra Pihlström^1, 2^, Riikka E. Mäkitie^1, 2, 5^, Emilie Dambroise^6^, Laurence Legeai-Mallet^6^, Pertti Panula^3^, Outi Mäkitie**^1, 2, 7, 8^ and Minna Pekkinen**†^1, 2, 7^

**Supplementary Table S1. *sgms2a* and *sgms2b* transcripts.**

|  |  |  |  |  |  |  |
| --- | --- | --- | --- | --- | --- | --- |
| **Transcript ID** | **Name** | **bp** | **Protein** | **Biotype** | **UniProt Match** | **Flags** |
| ***sgms2a*** |  |  |  |  |  |  |
| ENSDART00000141476.4 | *sgms2a-203* | 1773 | 351aa | Protein coding | A0A8M1NT99 | Ensembl Canonical,  APPRIS P1 |
| ENSDART00000143398.2 | *sgms2a-201* | 738 | 174aa | Protein coding | - | CDS 3' incomplete |
| ENSDART00000138301.2 | *sgms2a-202* | 713 | 143aa | Protein coding | - | CDS 3' incomplete |
| ENSDART00000133392.3 | *sgms2a-204* | 555 | No protein | Processed transcript | - | - |
|  |  |  |  |  |  |  |
| ***sgms2b*** |  |  |  |  |  |  |
| ENSDART00000053571.6 | *sgms2b-201* | 1481 | 373aa | Protein coding | Q6DEI3 | Ensembl Canonical,  APPRIS P1 |
| ENSDART00000149464.2 | *sgms2b-202* | 1005 | 312aa | Protein coding | - | CDS 3' incomplete |

Abbreviations: aa, amino acid; bp, base pair. Source: Ensembl genome assembly: GRCz11 (GCA_000002035.4)).^1^


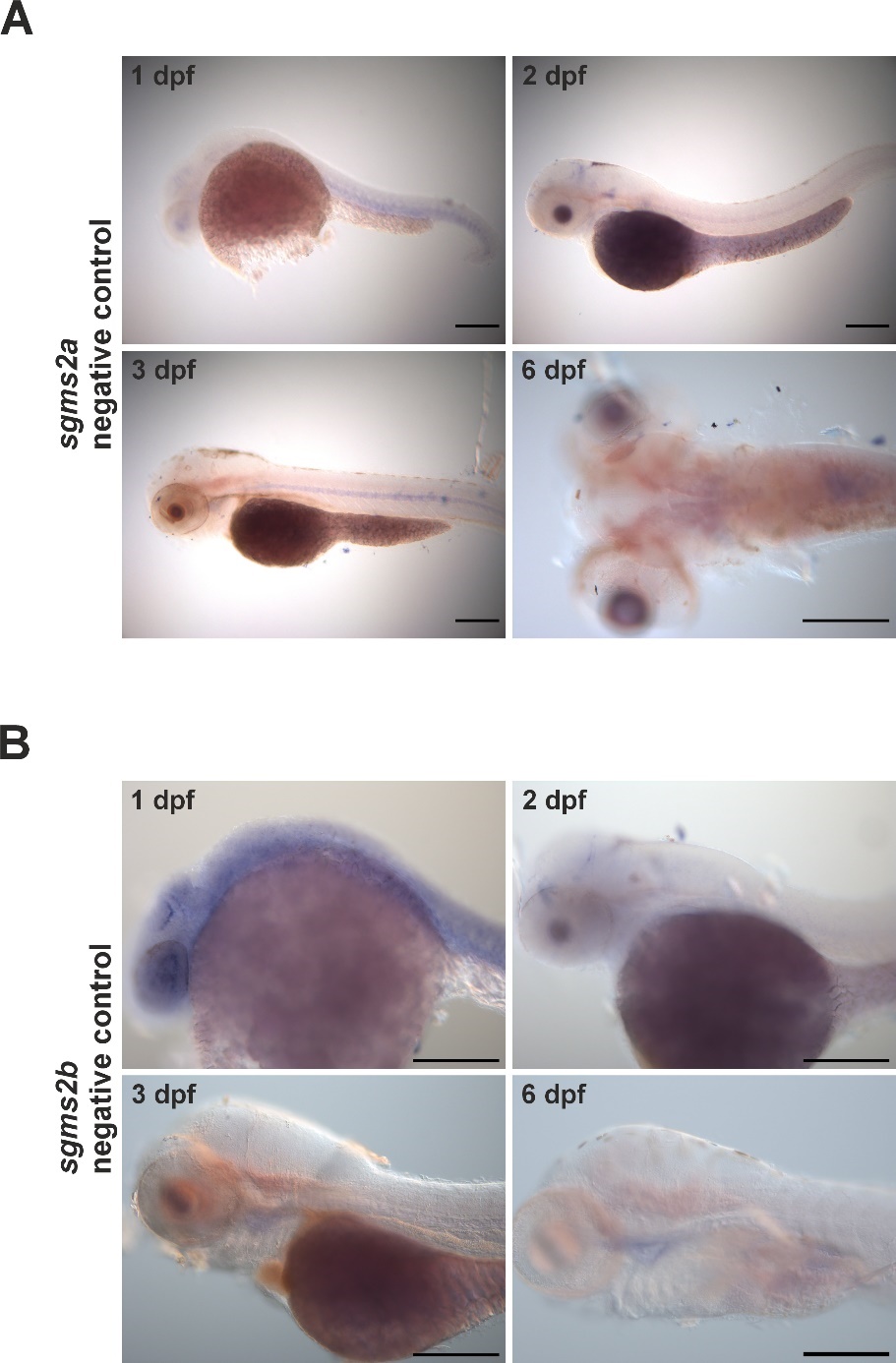


**Supplementary Figure S1. Whole-mount *in situ* hybridization in wild-type zebrafish embryos and larvae using sense probe as a negative control.** (A) Whole-mount *in situ* hybridization performed on 1, 2, 3, and 6 dpf WT embryos and larvae using the *sgms2a* sense RNA probe as a negative control. 1, 2, and 3 dpf are lateral views and 6 dpf is dorsal view. (B) Whole-mount *in situ* hybridization performed on 1, 2, 3, and 6 dpf WT embryos and larvae using the *sgms2b* sense RNA probe as negative control. 1 dpf embryo showed some background staining. 1, 2, 3, and 6 dpf are lateral views. Scale bar = 200 µm.


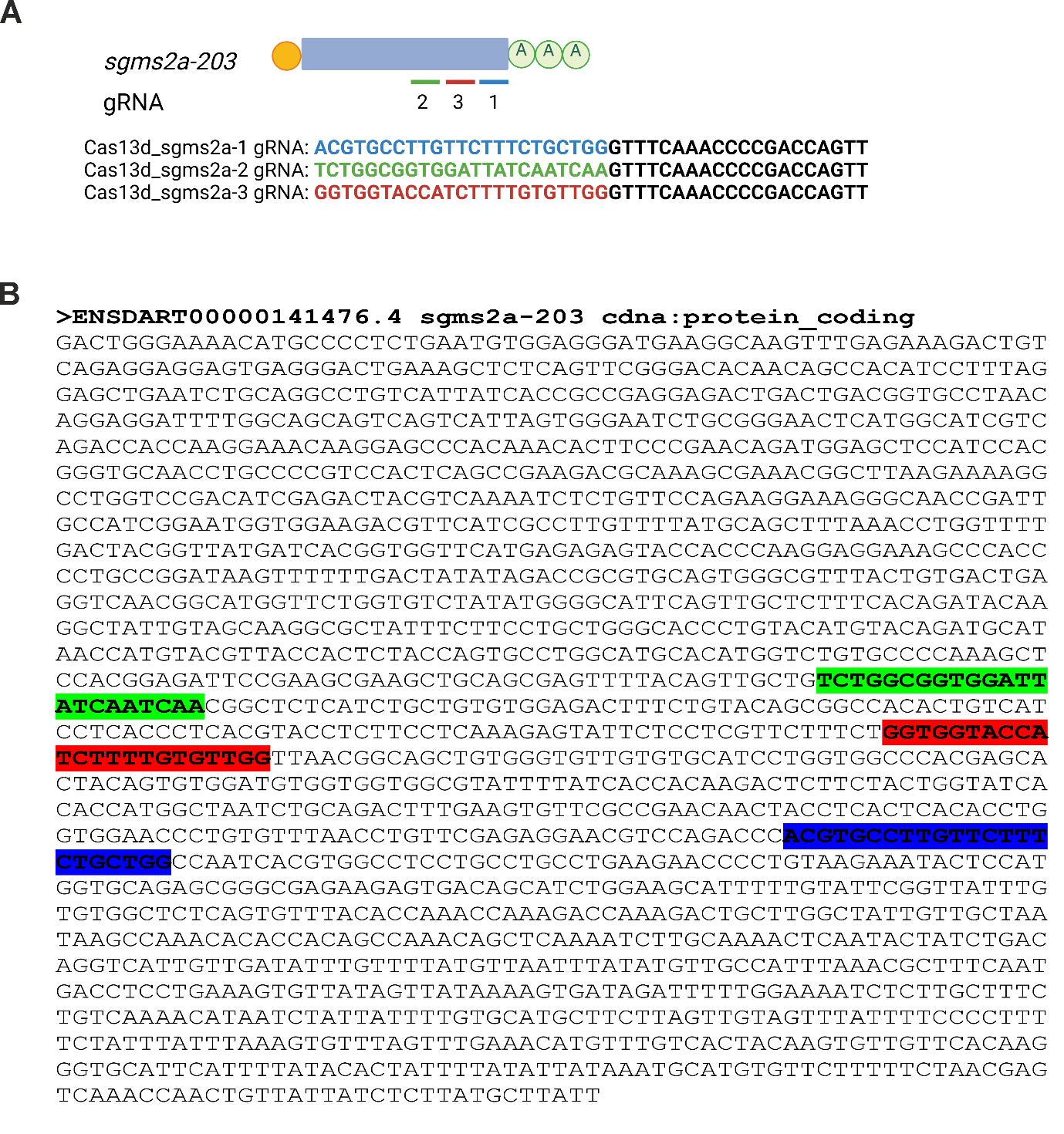
**Supplementary Figure S2. *sgms2a-203* transcript and Cas13d targets.** (A) Schematic illustration of *sgms2a-203* transcript with three marked gRNA targets. (B) *sgms2b-201* transcript (ENSDART00000141476.4) sequence (1773 bp) with marked three gRNA targets.


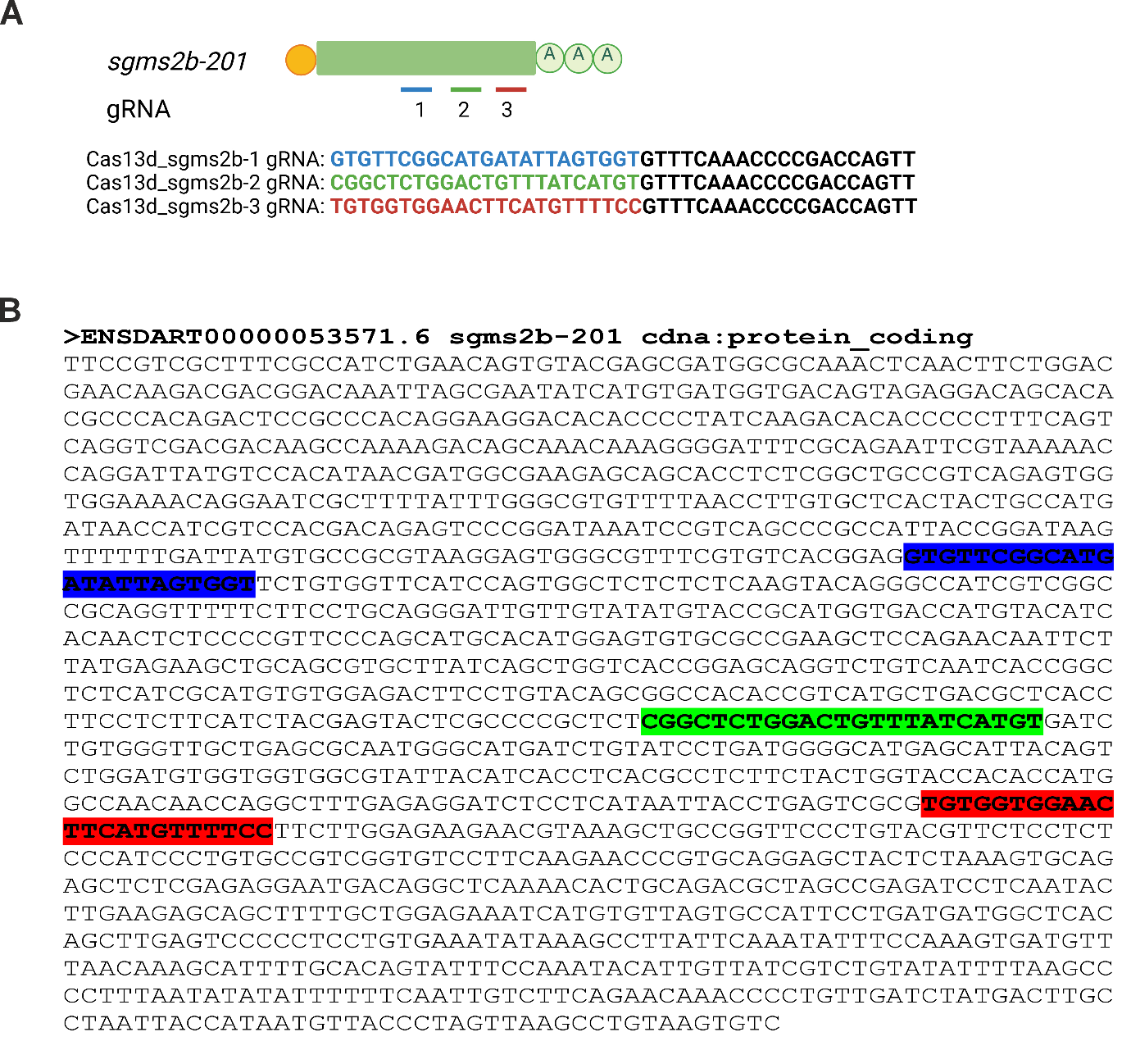
**Supplementary Figure S3. *sgms2b-201* transcript and Cas13d targets.** (A) Schematic illustration of *sgms2b-201* transcript with three marked gRNA targets. (B) *sgms2b-201* transcript (ENSDART00000053571.6) sequence (1481 bp) with marked three gRNA targets.


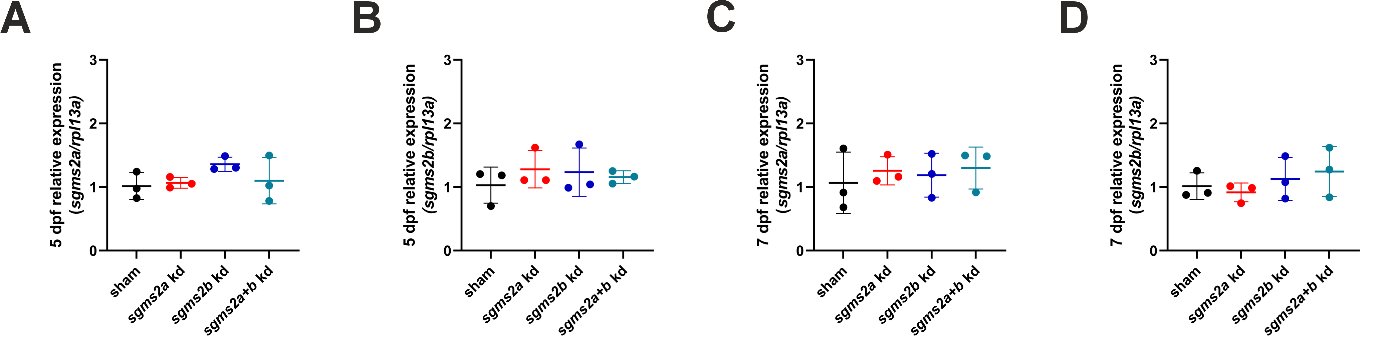


**Supplementary Figure S4**. ***sgms2a* and *sgms2b* mRNA expression in CRISPR-Cas13d-knockdown zebrafish larvae by RT-qPCR**. Expression of *sgms2a* and *sgms2b* mRNA at (A-B) 5 dpf and (C-D) 7 dpf sham, *sgms2a* kd, *sgms2b* kd, and *sgms2a+b* kd zebrafish larvae by RT-qPCR. Relative expression to *rpl13a*. Data is shown as scatter dot plots with mean ± SD. The results from three independent experiments were combined. The *sgms2a* kd, the *sgms2b* kd, and the *sgms2a+b* kd larvae were compared with the sham larvae (ordinary one-way ANOVA for multiple comparisons). *P*<0.05 was considered statistically significant. Only significant *p*-values were presented.

**References**

1. Dyer SC, Austine-Orimoloye O, Azov AG, et al. Ensembl 2025. *Nucleic Acids Res*. 2024;53(D1):D948-D957. doi:10.1093/nar/gkae1071
